# Testing a multi-behavioral intervention to improve oral health behaviors in the pediatric dental surgery population: protocol for the PROTECT trial

**DOI:** 10.3389/fpubh.2025.1488910

**Published:** 2025-01-22

**Authors:** Joanna Buscemi, Teresa G. Borowski, David Avenetti, Lisa Tussing-Humphreys, Molly Martin, Marc S. Atkins, Teresa Marshall, Michael Berbaum, Helen H. Lee

**Affiliations:** ^1^Department of Psychology, DePaul University, Chicago, IL, United States; ^2^Institute for Health Research and Policy, University of Illinois Chicago, Chicago, IL, United States; ^3^Department of Pediatric Dentistry, University of Illinois Chicago, Chicago, IL, United States; ^4^Department of Kinesiology and Nutrition, University of Illinois Chicago, Chicago, IL, United States; ^5^University of Illinois Cancer Center, University of Illinois Chicago, Chicago, IL, United States; ^6^Department of Pediatrics, University of Illinois Chicago, Chicago, IL, United States; ^7^Institute for Juvenile Research, University of Illinois Chicago, Chicago, IL, United States; ^8^Department of Preventive and Community Dentistry, University of Iowa, Iowa City, IA, United States; ^9^Department of Anesthesiology, University of Illinois Chicago, Chicago, IL, United States

**Keywords:** severe early childhood caries, community health worker, behavioral intervention, parenting intervention, oral health

## Abstract

Severe early childhood caries (S-ECC) is a common disease within marginalized pediatric populations. S-ECC is often treated under general anesthesia to facilitate extensive treatment in young children, but treatment does not address etiology of an infectious disease that is rooted in health behaviors. Without behavior changes related to toothbrushing and sugar consumption, many children experience recurrent disease, and some require subsequent surgeries. To improve post-surgery oral health, we developed PROTECT (Preventing Recurrent Operations Targeting Early Childhood Caries Treatment), a community health worker (CHW)-delivered behavioral intervention for caregivers that focuses on children’s oral health behaviors. This study aims to test the efficacy of the PROTECT intervention compared to Usual Care (UC), to improve behavioral oral health outcomes. We will conduct a randomized clinical trial to test the efficacy of PROTECT (*n* = 210) compared to UC (*n* = 210) in the pediatric DGA (dental surgery under general anesthesia) population. We developed PROTECT through an iterative process, incorporating feedback from caregivers, dentists, and community health workers and through a small pilot trial. Caregivers will be recruited at their dental clinic and then will engage in a 10-session intervention with a community health worker. Topics covered will include education about toothbrushing and sugar consumption, behavioral strategies (e.g., goal setting, problem solving, self-monitoring), positive parenting and stress management and maintenance. Our two primary outcomes are parental assisted toothbrushing (twice/day, 2 min each time) and reduced added sugar consumption (less than 10% of overall daily caloric intake). Proposed mechanisms of change are self-efficacy and positive parenting. The ultimate goal of PROTECT is to prevent subsequent surgical events for children presenting with S-ECC to prevent further chronic disease and reduce costs and stress for families who already experience high levels of systemic barriers to their health.

## 1 Introduction

### 1.1 Background

Dental caries is the most common chronic disease of early childhood, disproportionately affecting children who have been systemically oppressed [ethnic/racial minority groups, families of low-income, and those who live in rural areas (1, 2, 53)]. Young children who have poor oral health behaviors (e.g., inadequate tooth brushing, diet high in added sugar) are at risk for developing severe early childhood caries (S-ECC), which is an indication for dental surgery (3–5). Prevalence of S-ECC has declined and utilization of preventive dental care has increased over time (6). However, inequities in disease burden persist, and demand for dental surgery under general anesthesia (DGA) is increasing (1, 6). The impact of S-ECC on a child’s health ripples out across systemic and psychosocial well-being, with links to childhood obesity and oral health quality of life (7–10). Surgical events have inherent safety risks with the potential for iatrogenic harm (11, 12). Further, surgical intervention is expensive and ineffective in the long term (13, 14). Because surgical intervention does not directly address the etiologic factors, which are largely behavioral, approximately 50% of children have recurrent disease within 12 months after DGA (15). Dental surgery is risky, expensive, and not effective for S-ECC treatment in the long term (12, 13, 15, 16). To reduce the demand for surgery, we need to better understand how to change the exposures and behaviors that lead to becoming dental surgery patients (17, 18). Given that parenting behaviors influence a child’s oral health status, caregivers are an important catalyst for facilitating child behavior changes (19, 20). Positive parenting, such as appropriate monitoring of a child during tooth brushing or negotiating conflicts when children want sugary snacks, influences child health behaviors (21).

Tertiary prevention among young children with S-ECC, with a focus on parenting behaviors related to oral health, is essential to slowing disease progression and preventing future surgeries. Because behavior patterns established in early childhood tend to persist into adulthood (e.g., tooth brushing, dietary habits), early intervention is not only warranted, but potentially the most cost-effective, when targeted to parents of young children in the high-risk surgical population (22). While other studies have focused on primary prevention [e.g., (23)], ours focuses on a pediatric population that already has oral health disease.

### 1.2 Preliminary work

Our team conducted preliminary qualitative research with caregivers while their children were undergoing DGA and found that parenting behaviors contribute significantly to poor oral health behaviors (24). Specifically, caregivers reported offering a sugary snack to avoid a tantrum or scolding their child when they did not brush their teeth. Findings from this study suggested possible targets for intervention such as toothbrushing routine and supportive parenting techniques. This preliminary work, as well as other supportive studies, identified several barriers to changing oral health behaviors, including parenting style, dental self-efficacy, and oral health knowledge (24–26). This previous work identified the need for interventions to change oral health behaviors for the surgical population that are evidence-based, supportive, educational, responsive to health literacy, and adaptive to various psychosocial factors and household dynamics.

In order to change oral health behaviors such as tooth brushing and sugar consumption, a 6-month parenting intervention called Preventing Recurrent Operations Targeting Early Childhood Caries Treatment (PROTECT) was developed. The goals of PROTECT are to reduce S-ECC and DGA by providing parents with evidence-based support regarding toothbrushing, sugar consumption, and parenting during the 6-month postoperative period. A trained community health worker will deliver the intervention and provide additional resources to caregivers and families.

Our primary outcomes (tooth brushing frequency and % total calories from added sugar) are associated with S-ECC and have been identified as predominant behavior challenges for surgical families (3, 22, 27). PROTECT will be delivered by trained community health workers (CHWs) who have social proximity to our participants: CHWs have shared experiences and an understanding of clients and clients’ communities, which reduces stigma and aligns services with community norms (28–30). PROTECT will be delivered over a six-month interval beginning at the surgical event. This time period coincides with when many parents report high motivation to change behaviors and improve oral health (21, 24). Behavioral parenting interventions have been validated in mental health and childhood obesity, and we believe will impact S-ECC (31–34).

In our formative phase, we conducted semi-structured interviews with key constituents (dentists, CHWs, and caregivers of children undergoing DGA) to identify the appropriateness of the content and timing of the proposed 6-month parenting behavioral support program. Qualitative analysis of the interview transcripts determined that the PROTECT program was wanted, needed, and seen as acceptable by dentists, CHWs, and caregivers in the pediatric surgical population. Barriers to behavioral change were identified (e.g., multigenerational caregiving and caregiver discord, social determinants of health, incomplete health knowledge, and caregiver resistance to change). Our formative work also identified that caregivers require flexibility in intervention fidelity to maintain engagement (e.g., delivery of intervention content in a different order based on families’ immediate needs). We adjusted the program content and schedule to address barriers and increase engagement based on what we learned prior to conducting a pilot study to test feasibility with 12 caregivers. Of the 7 participants who completed the study, all reported twice a day toothbrushing and < 10% added sugar intake from total calories.

### 1.3 Purpose and aims

The purpose of this paper is to present the design of PROTECT, a randomized controlled trial (RCT) to test the efficacy of PROTECT (*n* = 210) compared to Usual Care (UC) (*n* = 210) in the pediatric DGA population. We hypothesize that participants in the PROTECT group will increase tooth brushing and decrease added sugar intake to a greater degree than those in the UC group. Assessments will occur at the time of dental surgery, 6-months post-surgery, and 6 months after intervention completion (i.e., 12 months post-surgery). We also aim to determine the mechanistic role of behavioral change targets in influencing intervention effectiveness. Per Social Cognitive Theory (28), we will estimate a mediation model with positive parenting and self-efficacy as mediators in the pathway to behavioral change (i.e., increases in positive parenting and self-efficacy leading to positive behavioral change—increases in toothbrushing and decreases in added sugar consumption).

## 2 Methods

### 2.1 Study design and setting

This study aims to test the efficacy of the PROTECT (Preventing Recurrent Operations Targeting Early Childhood Caries Treatment) intervention compared to Usual Care (UC) to improve behavioral oral health outcomes. We propose conducting a single-site Stage II (35) two-arm randomized controlled trial, which tests the efficacy of the PROTECT intervention delivered by community health workers (CHWs) for the pediatric DGA (dental surgery under general anesthesia) population. This is a prospective, individually randomized group treatment trial that will implement a behavioral parenting intervention (PROTECT) that starts at the time of DGA, including 10 sessions over 6 months, and will follow up for any changes in outcomes six-months post-intervention. Primary outcomes include tooth brushing frequency and percentage of total daily calories derived from added sugars; mechanisms of change will also be examined. Dental clinic providers will be blinded to randomization of participants.

### 2.2 Sample size determination

The sample size calculation was based on a two-arm parallel design for evaluating PROTECT versus UC effects on percent calories from added sugar intake and frequency of tooth brushing. We account for cluster sizes of 41–48 children per CHW in the intervention arm and assume independent observations in the control arm. Adjusting for retention of 85% at 12 months, the cluster size will be 48–56 participants per CHW in the intervention arm. The mean % energy contributed by added sugars is 14.3, SD = 10.7 among 2–8-year-old according to NHANES 2009–2012 data (36). It is possible that there are demographic differences between a nationally representative study and the primarily Medicaid-enrolled patients who present to UIC. We acknowledge that basing a sample size upon NHANES will likely underestimate added sugar intake and overestimate brushing frequency. However, our estimates will bias to a larger sample than needed, which is preferred. To our knowledge, there are no estimates related to our primary oral health behavior outcomes in the pediatric DGA population, which simply highlights the understudied nature of pediatric oral health. The sample size calculation was based on formula from Campbell and Walters (37) and Ahn et al. (38) implemented in PASS 15 software (39). The power calculation is based on group difference at the end of the intervention period relative to any difference at the baseline that might not be fully controlled by randomization. It is formalized as an additional change (increase or decrease) in the intervention arm relative to any change in the control arm (Group x Time interaction). The calculation takes into account partial clustering due to participants clustered in CHWs in the intervention arm only. We assume a 0.01–0.02 intra-class correlation coefficients due to CHW clustering, yielding sample size ranges. The significance level alpha was adjusted by Bonferroni correction to account for our two primary outcomes. With equal group allocation and a two-sided significance level of alpha = 0.025 (it is a conservative assumption given the hypothesized improvement in the primary outcomes in the PROTECT arm), we will target our intervention to bring the participants to the recommended guideline of 10% calories from the added sugars in the PROTECT arm (40). Hence, to detect a 4.3% change in calories from added sugars at 12 month follow-up (6-month post intervention) with 0.85 power, we would need 164–196 participants in each arm. Taking into account 85% retention rate at 12 month follow up (41), we will need to recruit 386–462 participants across two arms. Twice a day brushing frequency is 55% among high-risk toddlers in Chicago (42). The sample size calculation utilized test for difference in two proportions with unpooled standard deviations (43). The formula was adjusted to account for clustering in the intervention arm whereas the usual care arm assumed independent observations. We assumed equal group allocation and a two-sided significance level of alpha = 0.025. We assumed 0.01–0.02 intraclass correlation coefficients for the partial clustering effect. To detect 20% increase in twice a day brushing frequency in the PROTECT arm, bringing it to 75%, with 0.85 power, we will need 316–365 participants across two arms. Taking into account 85% retention rate at 12-month follow up (6 months post-intervention) (41), and combining sample size estimates from the two outcomes, we will recruit 420 participants across two arms (midpoint of higher range).

### 2.3 Study population and procedures

Participants will be caregivers of children who are presenting for dental surgery under general anesthesia at the dental clinic. The clinic currently provides dental surgery to healthy children in an office location in the College of Dentistry at the University of Illinois Chicago (UIC). The clinic schedules ~27–30 children for dental surgery every week, totaling ~1,200 surgical events per year. We plan to recruit 420 families over the course of 2–4 years.

In order to be eligible to participate in this study, an individual must be a caregiver of a child patient 7 years old or younger (≤ 96 months of age at the time of enrollment) scheduled for dental surgery under general anesthesia at the UIC clinic. Caregivers must meet all of the following criteria:

- caregivers are in same household >50% of the week;
- caregivers are English or Spanish-speaking,
- caregivers are aged ≥18 and < 90 years;
- caregivers have access to a computer or a telephone; and
- caregivers are willing to return for a routine preventive dental visit, per dentist recommendations as part of standard practice, 6 months after dental surgery at one of two dental clinics in our system.

This study will include individuals who are not yet adults (i.e., child dental surgical patients) and are too young to provide assent. With parental consent and permission, we will collect clinical information from children’s dental chart. If the caregiver is not the legal guardian, we will seek consent from the legal guardian.

An individual who meets any of the following criteria will be excluded from participation in this study:

- caregivers of foster children (the psychosocial environment and relationship dynamics of foster families are beyond the scope of this intervention);
- families who are planning to move out of state within the six-month period or are unwilling/unable to return to UIC for follow up dental visits during the study period;
- children with systemic health issues, as classified by American Society of Anesthesiology Classification ≥3, such as congenital anomalies, craniofacial syndromes, or severe systemic disease, as medical complexity is associated with other issues that influence a child’s health behaviors and caregiver-child interactions;
- children with developmental disorders (such as autism or developmental delay) that may impair their ability to perform routine oral health behaviors;
- people who are incarcerated;
- adults unable to consent (e.g., unable to read and/or understand the consent form through reading and discussion).

### 2.4 Recruitment

Recruitment is expected to begin in January of 2025. We plan to use multiple recruitment strategies. First, as part of standard care at the UIC Pediatric Dental Clinic, patients are required to visit the clinic prior to scheduling the pediatric dental surgery. All patients will see an informational poster in the waiting area of the dental clinic. At this appointment, potential participants—children who are determined to be potential surgical patients and their caregivers—will be directed to review a large poster (English and Spanish) that will explain the study rationale and eligibility criteria posted in the patient waiting area of the UIC Pediatric Dental Clinic. Informational recruitment flyers (English and Spanish) may also be provided. The informational recruitment flyer, its content, and a link to an eligibility screening and contact form (English and Spanish) will also be published on the College of Dentistry website. The flyers and poster will direct those who are interested or have questions to contact the study team via email or by scanning a QR code that leads to an eligibility screening and contact form, which includes an option to be put on a no-contact list if a patient decides not to participate. Researchers will follow-up with interested potential participants to set up a virtual screening/enrollment visit. Potential participants will be able to take the time needed to review the documents and make a decision about enrollment before scheduling this visit.

Second, if the patient is identified as a surgical candidate during the pre-surgical visit, a member of the research team will screen for eligibility based on child age and caregiver language of preference. Eligible patients may be approached in the clinic by the member of the research team and provided the recruitment flyer, additional information about the study, and an option to be put on a no-contact list. The research team member may describe the study procedures, ask further screening questions to determine eligibility, and discuss the benefits/risks of research activities, as well as provide a copy of the informed consent document for interested patients to review. The research team member will obtain and document verbal consent from the potential participant to schedule a study enrollment visit for individuals who are eligible, interested, and available for the duration of the study. Potential participants will be given sufficient time to review materials, and research team members will follow up to enroll interested participants.

Third, when an eligible patient is put on the surgical schedule (after the pre-surgical visit described above), research team members will review the scheduled surgical patients and screen for child age and language eligibility. Select members of the research team have access to the dental electronic record-keeping and scheduling software used by the College of Dentistry. These members will identify potential participants (i.e., English- and Spanish-speaking caregivers of children who are scheduled for dental surgery) for recruitment. These patients would have been exposed to the recruitment poster and flyer during their pre-surgical visit and had the opportunity to be put on a no-contact list. Members of the research teams may reach out to caregivers prior to the day of surgery to discuss the voluntary research opportunity, provide information about the study and procedures, and provide a copy of the informed consent document for interested patients to review. Potential participants will be given sufficient time to review materials, and research team members will follow up to enroll interested participants.

During the enrollment visit, a research team member will complete screening by verifying inclusion and exclusion criteria. Once full eligibility status is determined, the consent document will be discussed. Informed consent will be obtained and documented at this time. If not in-person, consent will be obtained through an online link to a consent form on REDCap (Research Electronic Data Capture). REDCap is a secure, web-based application designed to manage data collection for research studies that is primarily used for building and managing online surveys and databases, particularly in clinical, academic, and scientific research settings.

Baseline data may also be collected at this time, in which case the research team member would obtain self-reported data via verbal questionnaires; this practice is common and helpful for populations with low literacy levels. Data collected would include demographic information, oral health behaviors, parenting style, and nutrition/dietary habits (Caregivers will be compensated for the data collection visits as follows: $45 for time 1 and 2 and $55 for time 3).

We have made some important changes to our recruitment protocol to improve our recruitment rates. For example, we created a new position of a clinical research coordinator who will be on site and will be contacting every potential participant. For the pilot, we were dependent upon our study staff availability. Additionally, we have worked on branding online to promote trust with participants. Finally, we are recruiting before the time of surgery, rather than during so that the participants are not approached during the child’s surgical event.

### 2.5 Measures

Potential participants will be given a chance to review the informed consent and complete an eligibility screening form to ensure they meet the study’s inclusion/exclusion criteria prior to enrollment. Eligible, interested persons will then complete the enrollment process and be given the opportunity to complete the first data collection visit immediately or schedule it prior to or on the day of surgery. Participants in the intervention arm will complete baseline data collection prior to receiving the intervention.

Data in both arms will be collected during 3 visit periods: at baseline (around time of surgery), 6-months following surgery, and 12-months following surgery. Table 1 outlines what data will be collected at each time-point. Following are descriptions of each assessment.

**Table 1:** PROTECT data collection schedule.

Procedures	Enrollment	T1 Data	Surgery	T2 Data	T3 Data
Month	−3 to 0	-3 to 0	0	6	12
Eligibility criteria	X				
Signed consent form	X				
Demographics		X			
Child and caregiver BMI		X		X	X
Child clinical outcomes			X	X	X
Child dietary intake (NDSR)		X		X	X
Child oral health behaviors		X		X	X
Parent self-efficacy		X		X	X
Parenting (MAPS)		X		X	X
Feasibility, acceptability				X	
Study-exit					X

Demographics: We will collect child and caregiver race/ethnicity, date of birth, height and weight (to calculate BMI), and dental insurance status, as well as caregiver marital status, education, occupation, household income, household structure and size, and caregiver language preference. We will also ask for contact information for the parent and two other family members for tracking purposes (i.e., to facilitate continued contact and retention).

#### 2.5.1 Primary outcomes

Our primary outcomes include tooth brushing frequency and percentage of total calories derived from added sugars. To assess toothbrushing frequency and other child and caregiver brushing behaviors, 11 items from the Basic Research Factors Questionnaire (BRFQ) will be used. The BRFQ is a validated questionnaire to assess dental knowledge, attitudes, and behaviors of caregivers with young children. We will also assess self-reported frequency and length of brushing, assistance with brushing, and use of fluoridated toothpaste. The BRFQ is validated in English and has been translated into Spanish (not yet validated) by members of the research team.

To assess percentage of total calories derived from added sugars, we will conduct a 24-h dietary recall interview at baseline, the 6-month follow-up, and the 12-month follow-up. The child’s dietary intake from the previous day (12:00A–11:59P) will be captured in-person or via telephone or zoom using Nutrition Data System for Research (NDSR) data capture and analysis software. The software uses interview prompts to conduct a standardized multiple pass 24-h dietary recall. The multi-pass approach enables respondents to recall foods and beverages consumed with greater accuracy. The caregiver will be asked to use the food amounts booklet to aid the diet interview. A bilingual team member will use the Spanish interviewer prompts provided as an option in the NDSR system for all recalls that are conducted in Spanish. Data collection staff will be trained to conduct dietary recalls. Dietary recall data will be used to calculate nutrient intake (e.g., kcal, fat, protein, carbohydrate) and % kcal from total sugars and added sugars. The dietary recall interview is validated in both English and Spanish.

#### 2.5.2 Clinical outcomes

The Decayed, Missing, Filled Teeth Index (DMFT) will be used to assess disease severity for primary and any permanent teeth. Scores range from 1 to 20 if in the primary dentition. Receipt of urgent or emergent dental care or sedation or caries will also be documented. Select members of the research team will extract data from electronic dental records after a child has DGA or a preventive dental visit and enter into REDCap. Dental residents who will be trained to enter data into electronic records will be enteing the data. We will check the accuracy for 1/20 of the records for quality assurance. We will use this data to calculate the dmft/DMFT score, which will be stored in REDCap.

#### 2.5.3 Mechanisms of behavioral change

In addition to subscales in the above-described BRFQ, two additional assessments will be used to measure mechanisms of change, including self-efficacy and positive parenting. The Self-Efficacy Scale for Maternal Oral Care (SESMO) was designed for mothers of children up to 8 years old. It consists of 12 items (on a 4-point Likert scale), divided into two self-efficacy domains (subscales): (i) self-efficacy for tooth brushing and (ii) self-efficacy for dietary habits. This measure has been validated in English and Spanish. The Multidimensional Assessment of Parenting Scale (MAPS) measures parenting practices and includes measures of positive and negative dimensions of warmth, hostility, and behavioral control. It includes 34 items on a 5-point Likert scale and has been validated in English and translated into Spanish (not yet validated) by members of the research team.

#### 2.5.4 Intervention-specific data

Participating caregivers in the intervention arm will also complete measures of Acceptability and Feasibility, as well as a verbal intervention-exit interview to discuss their thoughts on the program. Feasibility will be measured via recruitment and retention rates, and engagement in intervention sessions. A 70% recruitment rate and 80% retention rate will be considered adequate. Completion of 80% or more of the available sessions will be considered adequate for engagement. In addition, participants will complete a validated self-report measure of intervention feasibility. Above average scores (3 or above on a 5-point scale) will be considered acceptable. Participants will also be asked to complete a validated acceptability measure assessing usefulness and satisfaction of the intervention (54). Above average scores will be considered acceptable (3 or above on a 5-point scale). The intervention-exit verbal interview contains items assessing specific intervention components of PROTECT to identify which components were most helpful and led to acceptability of the program. Questions also address barriers to and facilitators of behavioral change.

#### 2.5.5 Data collection

All questionnaire and survey data will be collected by trained research team members through participant phone, zoom, or in-person visits and directly entered into REDCap, a secure web application used by UIC for managing surveys and databases that can be used to collect any type of data in compliance with HIPAA.^1^ NDSR dietary data is stored on the NDSR software on a password-protected, encrypted computer. Clinical data related to the child’s oral health will be collected from dental records and stored in REDCap.

All measures received by both arms will be collected by Research Assistants (RAs). To maintain RA blinding at Time 2 and Time 3, the acceptability, feasibility, and intervention-exit questionnaires will be sent via survey link to be completed by participants. The Project Manager will receive an alert—programmed into REDCap—once Time 2 forms are completed by the RA and send the final intervention-specific forms via survey to participants. Blinding for the primary outcomes (toothbrushing and diet) remains the priority over potentially missing feasibility data.

### 2.6 Randomization

**Figure 1:**
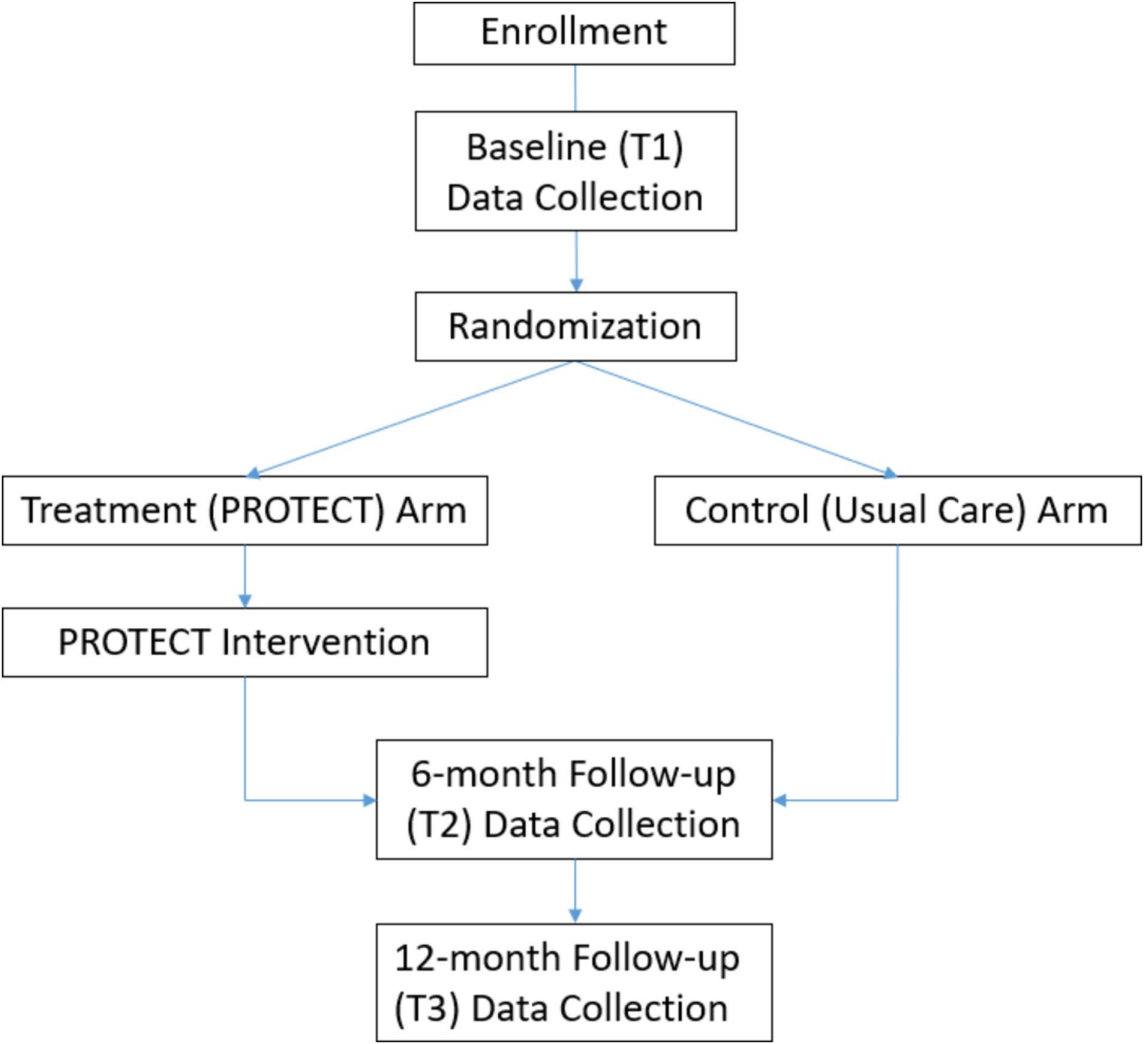
Participant flow throughout the study.

Following the baseline assessment, participants will be randomized to one of two arms: (1) PROTECT (intervention) or (2) Usual Care (UC; control). A random allocation table with 4 and 6 block sizes will be generated in SAS using pseudo-random number generator and implemented in the REDCap randomization module. Please see Figure 1 for participant flow throughout the study.

#### 2.6.1 Intervention

PROTECT is a 6-month parenting intervention that focuses on harnessing evidence-based parenting strategies to increase a child’s tooth brushing and decrease a child’s sugar consumption. The PROTECT intervention was developed by members of a scientific team based upon prior work, current evidence, and existing materials from oral health curricula (e.g., Oral Health Forum, Heartland Alliance) and CHW training curricula (e.g., Coordinate Oral Health Promotion Chicago). The intervention was further refined through formative interviews with dental providers, CHWs, and caregivers of children undergoing DGA. In addition to oral health and nutrition, sessions will cover topics such as positive parenting, goal setting, stress management, and problem-solving. The intervention will be delivered by community health workers (CHWs) who have social proximity to our participants, some who are fluent in Spanish. CHWs will be hired as part of the research team. CHWs will be paired with families with a child who is scheduled to have dental surgery at UIC. A CHW will work with each family to apply positive parenting skills to help their child consume less sugar and assist with daily toothbrushing. The CHW will meet (in person or remotely) with a caregiver over the six-month intervention program for 10 (five informational and five maintenance or check-in) sessions (see Table 2). Sessions will last 30–60 min and will address knowledge, application to daily life, and reflections on challenges to behavior change. During the second session, the CHW will review a Social Determinants of Health Needs Assessment with the caregiver to identify factors such as housing and food insecurity. The CHW is prepared to connect a caregiver to social agencies, public programs, and assist in finding a dental provider, at the request of a caregiver. During CHW training, the CHWs will receive resource sheets with programs and resources to address stress, mental health, immigration, Medicaid, navigation of the healthcare delivery system, safe housing and transportation, education and job opportunities, access to safe water and nutritious food, childcare, access to physical activity opportunities, and English language classes. Additionally, the CHWs will be trained and supported in identifying if a caregiver would be better supported by a trained therapist. In this situation, the CHWs will connect the caregiver to the social work team within the UIC pediatric dental clinic. This social work team is already integrated into the pediatric dental clinic in order to provide care coordination and therapy to clinic families.

**Table 2:** PROTECT intervention schedule.

Session #	Schedule	Session type	Session time	Session content
1	Baseline	In-Person (during child’s dental surgery) or virtual	60 min	Tooth health, toothbrushing, eating, and drinking
2	1 week	Phone (or in-person)	30 min	Parenting skills to support tooth health
3	2 weeks	Phone/Zoom	30 min	Rewards and routines
4	3 weeks	Phone/Zoom	30 min	Managing your emotions
5	4 weeks	Phone/Zoom	30 min	Monitoring and problem-solving
6	2 months	Phone/Zoom	30 min	Maintaining behavior change
7	3 months	Phone/Zoom	30 min	Maintaining behavior change
8	4 months	Phone/Zoom	30 min	Maintaining behavior change
9	5 months	Phone/Zoom	30 min	Maintaining behavior change
10	6 months	Phone/Zoom	30 min	Graduation session

The term “flexibility within fidelity” refers to an approach to intervention delivery that both honors the fidelity of the manual (e.g., the importance of closely following a manualized behavioral intervention) and the importance of flexibility within that model. CHWs will cover all of the topics included in this manual and keep track of what is introduced and covered with each participant using a checklist (recorded in an implementation diary in REDCap); however, delivery is not so rigid that we miss opportunities to present content when it arises (and thus damage participant engagement). For example, if a participant introduces a barrier to the CHW in session, the CHW is encouraged to engage in on-the-spot problem solving with the participant even if introduction of the problem-solving skills comes later in the manual. Based on interviews with caregivers and CHWs during formative work, allowing CHWs to deliver topics in variable order will translate to greater participant relevance and engagement. CHWs will make sure to cover all the checklist items and content with consistent messaging.

The control, or Usual Care (UC) group will receive usual clinical care, which consists of education during and immediately after surgery. Families randomized to the UC arm will receive the usual standard of care between the time they are identified as surgical candidates to the point when they are scheduled to have their post-surgical visit. Clinical education is provided by pediatric dental residents, and at least one pre-surgical visit is designed to allow families to discuss how their oral health behaviors contribute to caries and answer any questions regarding changing oral health behaviors. Families who are experiencing significant social issues which interfere with their ability to care for their child’s teeth are identified by clinic staff and referred to a full-time social worker employed by the dental clinic. The post-surgical visit typically occurs within 1–2 weeks after surgery and is intended to be a brief exam focused on determining if there are any complications related to the procedures (e.g., infection). Families randomized to the PROTECT arm will also receive usual clinical care. Select team members have access to records noting care deviations for participating patients.

### 2.7 Intervention fidelity and compliance

The CHW will complete an Implementation Diary following each session to track which and how much content was delivered to participants. Documentation after every encounter will record the date, curriculum topics covered, resources utilized, amount of time spent, and issues encountered after each visit. The Implementation Diary will track fidelity, adaptations, and adherence to the intervention protocol, as well as barriers related to behavior change. The Implementation Diary forms will be reviewed monthly by unblinded staff to ensure fidelity and also to inform the study team on areas of focus and challenge. The data will also be used in final analyses to determine “dose” of intervention and to assess the influence of specific topics/skills on outcomes.

The CHWs will be hired based on their knowledge of oral health. Prior to implementation, CHWs will be trained in PROTECT intervention delivery and study content and motivational interviewing, completing at least 3 practice sessions reviewed by our clinical psychologist to ensure competence and fidelity to the intervention. Fidelity will be monitored throughout the implementation, and additional training, guidance and support for CHWs will be available as needed based on fidelity assessments: Twenty percent of all intervention visits will be audio-recorded and reviewed by the clinical psychologist to assess fidelity of CHW competence using the Fidelity Assessment Form. Within the form, CHW skills (e.g., clarity of content, interpersonal effectiveness) are assessed on a scale from 1 to 5. An average score of <4 requires evaluation and possible remediation. Audio files will be immediately uploaded into REDCap and reviewed by the clinical psychologist monthly. Ongoing training for the CHWs will be provided through regularly monthly meetings with the clinical psychologist supervisor. Any audio files of PROTECT program sessions between a CHW and family will only be shared with the clinical psychologist on our team to assess CHW competence and fidelity to the intervention.

## 3 Statistical analyses plan

Our general statistical approach employs the Linear Mixed Model (LMM) and the Generalized LMM for categorical responses (44). These approaches accommodate both repeated measures in individual participants and clustering of participants. They are quite versatile and can be used for subject-specific analyses and, depending on distributional assumptions, marginal or population-averaged analyses. They also can be used to conduct both moderator and mediator analyses—the latter requiring multiple runs. Such models are available in SAS and R statistical packages; they can also be used within a structural equations modeling framework (e.g., lavaan and sem in R, or standalone Mplus). Bayesian estimation approaches are also available (rstan and brms in R). LMM and GLMM are widely accepted, standard approaches to longitudinal data analysis in modern statistical practice.

The primary outcomes are frequency of tooth brushing and percentage of calorie consumption from added sugar. These outcomes will be evaluated at baseline, 6 months post-surgery, and 12 months post-surgery. The main evaluation point is the 12-month (post-surgery) follow-up. We will examine baseline descriptive statistics for primary outcome as well as at each evaluation points. Descriptive statistics of demographic, child and caregiver characteristics, and proposed mechanisms of change will be calculated at the baseline by two groups. Frequency of tooth brushing is ordinal measure and to fully utilize the ordinal nature it will be analyzed by cumulative logistic regression with group as the main predictor. First, we will test for group differences at 12-month follow-up. We will follow up with mixed effect cumulative logistic model that will use all evaluations of the primary outcomes over time (45). Time by group interaction will be the main parameter of interest. Different variance–covariance structure, such as AR(1), Toeplitz, and unstructured, will be considered to fully account for repeated measurements. We will consider non-linear trend to explore diminishing effect of intervention over 6-month post intervention period. As a check for robustness of the primary analysis results, we will consider adjusting for covariates. In a similar manner, we will evaluate intervention effect on the second primary outcome with the exception of using statistical methods for a continuous measure. The added sugar outcome will be derived to determine percent of calories consumed from added sugars from the 24-h recall measure (NDSR) and is a continuous outcome. Specifically, the 6-month intervention effect will be evaluated with a t-test and a linear regression model will be used throughout. Residual diagnostic will be performed to check for deviations from normality. If considerable deviations are found, variable transformation will be attempted to bring original distribution close to normal. All statistical tests and models will adjust for factors used in stratified randomization. To control for multiple outcomes, the intervention effect will be declared significant at 0.025 level according with Bonferroni correction. Secondary outcomes will be analyzed following similar steps outlined above. These analyses will be conducted in SAS (v.9.4 or later). The analysis will be carried out under intention-to-treat (ITT) principles, implying that respondents who are randomized must be represented in analysis and therefore have missing data imputed. We will follow the approach of Little and Yau (46), whose approach to ITT conducts a sensitivity analysis to various missing data scenarios. Research assistants will assist with collecting data and will enter data into REDCap, hence we anticipate a very small fraction of missing data. We recognize possible clustering effect in the intervention arm due to CHW delivering the intervention. To account for the partial clustering, we will extend models to 3-level mixed-effect models with participants clustered in CHW as highest level of clustering. We will extend model with random slope for treatment effect (group) not random intercept, which amounts to random intercept in the intervention arm only (47). The model estimates ICC in the intervention arm only and ICC in the control arm is modeled to be zero. In a trial with CHW delivering an intervention in the treatment arm and usual care in the control arm we might expect participants to have different variability between arms. The model further can be extended to allow for heterogeneous variance. This model will be estimated in R. We will correct degrees of freedom with Kenward-Roger approximation to control for Type 1 error rate which could be inflated with few clusters (48).

To evaluate mechanism of change in primary outcomes as a result of intervention, we will estimate a mediation model with self-efficacy and positive parenting as mediators on the pathway of change. According to the Partners Achieving Student Success model informed by SCT (34), we hypothesize that intervention will affect our primary outcome through changes in caregiver self-efficacy or parenting strategies. Potential mediators will be evaluated longitudinally and their mediating effects will be evaluated one at a time using a longitudinal mediation model formulated via latent growth curve model (49, 50). The model will control for a rich set of variables on the mediator-outcome pathway. The model will be estimated in the Mplus structural equation program (51), which provides bootstrap-based tests of indirect and direct effects. We will also consider multiple sequential mediators in a single model, such as intervention will change caregiver’s self-efficacy, which will change their parenting strategy, which will result in more frequent tooth brushing and reduction of added sugar consumption.

### 3.1 Anticipated results

The described study’s central hypothesis is that PROTECT, a CHW-led behavioral intervention for caregivers, will lead to sustained changes in a child’s oral health behaviors in the post-surgical period. We anticipate significant differences between participants in the intervention vs. usual care arms in primary outcomes (increased proportion who brush twice/day; decreased total percent daily caloric intake from added sugars).

## 4 Discussion

PROTECT is a CHW-delivered behavioral intervention for caregivers that focuses on children’s oral health behaviors. The described study aims to test the efficacy of the PROTECT intervention compared to Usual Care (UC), to improve behavioral oral health outcomes. We will conduct a randomized clinical trial to test the efficacy of PROTECT (*n* = 210) compared to UC (*n* = 210) in the pediatric DGA (dental surgery under general anesthesia) population. We developed PROTECT through an iterative process, incorporating feedback from caregivers, dentists, and community health workers as well as through a small pilot trial. Caregivers will be recruited at their dental clinic and then half will engage in a 10-session intervention with a community health worker. Topics covered will include education around toothbrushing and sugar consumption, behavioral strategies (e.g., goal setting, problem solving, self-monitoring), positive parenting, and stress management. Primary outcomes include tooth brushing frequency and percentage of total calories derived from added sugars. Proposed mechanisms of change are self-efficacy and positive parenting. Strengths of PROTECT include our use of evidence-based behavioral and parenting tools to address difficulty to change behavior, the use of CHWs as the interventionists, the remote delivery and overall flexibility of the intervention, and the inclusion of assessing for and addressing common social determinants of health that are common in Medicaid-enrolled families.

There are several innovations in the current study. For example, we plan to target a population with the most severe disease to implement a CHW-led behavioral intervention in the time after dental surgery. We focus on surgical families because they have greatest potential to benefit and have previously expressed a desire for parenting support at the exact time of their child’s dental surgery (19). Children living with severe disease and presenting for DGA experience poor quality of life related to their caries [pain, difficulty chewing; (8, 9)]. Their parents are motivated to improve their child’s oral health and are receptive to help in changing behaviors (14). The surgical event is an ideal time to intervene, not only because parents are receptive to change, but also because they are already engaged in the health system as part of the DGA experience (14). Finally, we believe involving CHWs to implement our intervention will be a critical part of success. CHWs promote greater engagement and help mitigate barriers to health services faced by minoritized populations by leveraging their social proximity—relating to parents through shared similarities and experiences (e.g., understanding of culture, parenthood, life hardships), creating a sense of equality and “being on their level” (30).

The ultimate goals of PROTECT are to change the paradigm away from severe disease and treatment and toward promotion of oral health. Clinically, this may manifest as reduced rates of recurrent caries and subsequent surgical events and reduced costs and stress for families who already experience high levels of systemic barriers to their health. In the long term, it is our hope to assess the benefits of PROTECT and its impact on whole families (not just the identified patient) across the lifespan at the household level. We also hope to test the dissemination and implementation of PROTECT in community dental clinics to increase the reach and impact of this intervention for those who need it most.

## Data Availability

The original contributions presented in the study are included in the article/supplementary material, further inquiries can be directed to the corresponding author.

## Footnotes

^1^ https://www.project-redcap.org/
